# Low Food Security is Related with More Memory Complaints in a Clinic‐Based Sample of Latino Adults

**DOI:** 10.1002/alz.086595

**Published:** 2025-01-09

**Authors:** Joseph Saenz, Laura Tanner

**Affiliations:** ^1^ Arizona State University, Phoenix, AZ USA; ^2^ Arizona State Univeristy, Phoenix, AZ USA

## Abstract

**Background:**

Low food security has been linked with both diminished objective cognitive outcomes and lower subjective assessments of cognition in studies across the globe. Subjective memory complaints may be an important indicator of memory problems experienced in one’s daily life. However, few studies have explored links between low food security and subjective memory in Latinos, despite the higher prevalence of food insecurity in the Latino population. The aim of this study is to investigate potential links between low food security and subjective memory complaints in a sample of Latinos.

**Method:**

We used the Sangre Por Salud (SPS) data, a sample of Latino adults from a federally qualified community health center in Phoenix, Arizona who enrolled between 2013 and 2018. Individuals age 45+ (n = 1,041) are included in our analytic sample. Food security was assessed using the Household Food Security scale and memory complaints were measured using the Frequency of Forgetting scale. Potential links between low food security and subjective memory complaints were tested using linear regression controlling for demographic factors (age, gender, education, nativity), self‐rated health, and perceived social support.

**Result:**

Preliminary results indicated that 75% of the sample were highly/marginally food secure, 18% had low food security, and 7% had very low food security. Food security was more common in the US born and among those with higher social support. In multivariate analyses, compared to the highly/marginally food secure, low food security related with 0.26 standard deviations higher memory complaint (p<0.01) whereas very low food security related with 0.28 standard deviations higher memory complaint (p<0.05).

**Conclusion:**

Findings suggest an association between experiences of low food security and higher subjective memory complaints in Latinos. Subjective memory complaints are related with future cognitive impairment and dementia. Future studies should evaluate the potential cognitive benefits of interventions targeting food insecurity.

